# Five-year follow-up with the PreserFlo MicroShunt for open-angle glaucoma

**DOI:** 10.1038/s41433-025-03707-3

**Published:** 2025-02-20

**Authors:** Lotte M. J. Scheres, Stefani Kujovic-Aleksov, Bjorn Winkens, Ronald M. P. C. de Crom, Carroll A. B. Webers, Henny J. M. Beckers

**Affiliations:** 1 University Eye Clinic Maastricht, Maastricht University Medical Center +, Maastricht, the Netherlands; 2 Department of Methodology and Statistics, Care and Public Health Research Institute (CAPHRI), Maastricht University, Maastricht, The Netherlands

**Keywords:** Glaucoma, Surgery

## Abstract

**Purpose:**

To report on five-year results with the PreserFlo MicroShunt (MicroShunt) for the surgical treatment of open-angle glaucoma.

**Patients and methods:**

Retrospective case series of consecutive patients who underwent a stand-alone MicroShunt implantation at the University Eye Clinic of Maastricht. If a patient underwent the procedure in both eyes, only the first eye was included in the analysis. MicroShunt implantation was augmented with 0.2 mg/ml mitomycin-C. The primary outcome was intraocular pressure (IOP) during follow-up. Furthermore, information on IOP-lowering medication use, success rates, reoperation rates, and postoperative complications was collected.

**Results:**

Sixty-six eyes were included for analyses. Diagnoses included primary open-angle glaucoma (88%) and pigmentary glaucoma (12%). The majority of patients had moderate or advanced glaucoma, based on the mean deviation of the visual field examination. Mean (95% - confidence interval) IOP dropped from 21.8 (20.8–22.8) at baseline to 13.2 (11.8–14.6) mmHg after 5 years (*p* < 0.001). Mean number of IOP-lowering medications was reduced from 2.5 (2.2–2.9) at baseline to 0.9 (0.5–1.2), 1.0 (0.7–1.4), and 1.1 (0.7–1.5) after three, four, and five years (all *p* < 0.001). Needling or surgical revision was performed in twelve eyes (18%). Nineteen eyes (29%) required further IOP-lowering surgery. Postoperative complications were usually mild and self-limiting and included early hypotony, shallow anterior chamber, and hyphaema.

**Conclusions:**

After five years, the MicroShunt was found to be a safe procedure, leading to a sustained reduction in mean IOP and number of IOP-lowering medications. However, almost one third of the eyes required further IOP-lowering interventions.

## Introduction

Trabeculectomy remains the ‘gold standard’ as an initial surgical procedure for open-angle glaucoma [1]. It creates a fistula redirecting aqueous humour into the subconjunctival space, forming a filtering bleb. It is highly effective for lowering IOP, and there is ample experience with the procedure, which has, in turn improved its safety profile [2]. Nevertheless, intensive postoperative care is paramount in achieving successful results, especially during the first postoperative months [3–6]. Less invasive bleb-forming glaucoma surgical techniques have been commercially introduced to offer safe alternatives to traditional glaucoma surgeries. In theory, less invasive bleb-forming surgical procedures may lead to sufficient IOP reduction and achieve a target in the low teens, similar to trabeculectomy.

The PreserFlo MicroShunt (Santen, Osaka, Japan) (MicroShunt) uses a subconjunctival route for aqueous humour drainage [7]. The MicroShunt is an 8.5 mm long stent with a 70μm lumen made of a novel polymer, poly(styrene-*block*-isobutylene-*block*-styrene), ‘SIBS’. SIBS is biostable, thermoplastic, and provokes less inflammation than other commonly used materials [8, 9]. Its lumen design is based on the Hagen-Poiseuille equation to regulate aqueous flow and thus reduce the risk of early hypotony without the need for temporary tube occlusion [7].

Studies evaluating the efficacy of the MicroShunt have shown encouraging results, demonstrating effective IOP-lowering in patients with primary open-angle glaucoma (POAG) [10–12] and pseudoexfoliation glaucoma (PEXG) [13, 14]. Moreover, some evidence supports the use of MicroShunt in secondary and refractory glaucoma, underscoring its potential for managing more intricate glaucoma patients [15, 16]. Compared to trabeculectomy in a randomized controlled trial, the MicroShunt was shown to be less efficacious in terms of surgical success but with significantly fewer complications [17].

However, long-term evidence on this device remains limited to pioneering studies that helped define the MicroShunt and the implant procedure [10, 11, 18]. Long-term outcomes in real-world settings are required to further elucidate the long-term outcomes and potential complications associated with the MicroShunt. In the current study, we report on our five-year cohort of patients with open-angle glaucoma who underwent MicroShunt implantation augmented with Mitomycin C (MMC).

## Subjects and methods

### Patients

All patients who consecutively underwent a stand-alone MicroShunt implantation augmented with MMC, at least five years prior to data collection at the glaucoma clinic of the University Eye Clinic Maastricht, were included. Patients were eligible for surgery if they were inadequately controlled on maximum tolerated therapy and/or had progression of visual field loss and did not have a history of glaucoma surgery. If patients were lost to follow-up within the first three months after surgery, patients were excluded unless this was due to a re-intervention or an adverse event. If a patient underwent the procedure in both eyes during the study period, only the first eye was included in the analysis. The study (METC 2019—1172) was approved by the local ethics committee of the Maastricht University Medical Centre+ (Maastricht, The Netherlands) adhered to the tenets of the Declaration of Helsinki.

### Procedures

Almost all surgeries were performed by an experienced surgeon (HB). Sub-Tenon’s anaesthesia was chosen in all cases. The procedure has been described in detail elsewhere [19]. In brief, a fornix-based conjunctival flap was created, after which a deep sub-Tenon’s pocket was formed. To prevent bleeding, light cautery was applied to the sclera. A 0.2 mg/ml concentration of MMC was applied for 2–3 min. The scleral surface was rinsed thoroughly and marked 3 mm from the limbus. A scleral pocket was made with a triangular knife and the MicroShunt was placed through this pocket into the anterior chamber via a scleral needle track.

Following surgery, IOP-lowering medications were discontinued. Postoperatively, a topical unpreserved antibiotic (ofloxacin) (Trafloxal®, Bausch&Lomb Pharma, USA) was prescribed four times daily for two weeks. Topical anti-inflammatory therapy with unpreserved steroids (dexamethasone 0.1%) was started 4–6 times daily and was gradually tapered off over a period of several months according to bleb formation, inflammation, and wound healing response. In the event of an impending bleb failure, either bleb needling, open revision, and/or an additional IOP-lowering intervention was performed at the discretion of the treating surgeon. Needling and open bleb revisions were performed at the discretion of the operating surgeon. Open revisions were augmented with an antimetabolite (5FU or MMC 0.2 mg/ml) or Ologen® (Aeon Astron Europe B.V., Leiden, The Netherlands).

### Outcome measurements

The primary outcome was IOP measured with Goldmann applanation tonometry. Secondary outcomes included the number of postoperative IOP-lowering medications, success/failure rates, visual field progression, visual recovery rates, additional glaucoma interventions, and safety. The number of IOP-lowering medications were counted as the number of medication classes with oral acetazolamide as a separate group. Complete success was defined as an IOP 6–18 mmHg without IOP-lowering medication. If the IOP level was achieved with IOP-lowering medication, it was considered qualified success. Failure was defined as IOP level >18 mmHg or ≤5 mmHg at two consecutive visits after 3 months of follow-up, loss of light perception, or if additional glaucoma surgery was needed following MicroShunt. Bleb needling or open revision as such were not considered as failure. Success rates were also examined for an upper IOP level of 15 mmHg. As this study did not use preoperative washout IOPs, and preoperative IOP was already targeted as low as possible with maximum tolerated medication, incorporating a percentage of IOP reduction did not seem fit. Visual field progression was assessed with the Humphrey Field Analyser (HFA; Carl Zeiss Meditec, Jena, Germany). Visual acuity was measured using spectacle-corrected distance visual acuity (CDVA) in Snellen fractions and converted to the logarithm of the minimum angle of resolution (LogMAR). We considered visual recovery as achieved when CDVA was equal to baseline CDVA ± 0.15 logMAR, as limits of agreement for test-retest measures of visual acuity were shown to be about ±0.15 logMAR [20]. Surgical complications that occurred during the first three postoperative months were regarded as early postoperative complications, and late postoperative complications as those that occurred after three months. To evaluate hypotony, we used the numeric definition of a single measurement of IOP ≤ 5 mmHg after surgery regardless of concomitant complications, as suggested by the World Glaucoma Association (WGA) guidelines [21].

### Statistical analysis

Statistical analysis was performed using SPSS Statistics version 28 (IBM Inc., Armonk, NY, USA). Visits were collected from the electronic patient records using the time windows proposed by the WGA guidelines [21]. Visits outside of the time-windows were considered missing at random. Patients who required an additional IOP lowering intervention were included in the postoperative analysis up to the moment of the decision to intervene. Categorical data was presented as counts and percentages and continuous data as mean (95%- confidence interval (CI)) or mean ± standard deviation (SD). Linear mixed models (LMM) were fitted to investigate the longitudinal trend in IOP, medication-use, and visual field progression, where time (6 categories: preoperative, 1, 2, 3, 4, and 5 years) was used as a fixed factor and several covariance structures (including unstructured and heterogeneous first-order auto-regressive, Toeplitz or compound symmetry) were considered for the repeated measures, where the best model was selected based on the lowest Bayes Information Criterion (BIC). LMM were used as these incorporate all available data and assume missing at random, i.e. the data that are missing are related to the observed data, which then could be incorporated in the model. Estimated marginal means (EMM) and estimated mean differences with 95% confidence intervals (CI) were reported. Cumulative probability of failure (IOP above a pre-specified cut-off value (18 or 15 mmHg)) are presented using Kaplan-Meier (KM) curves.

## Results

Three eyes of the 86 eyes were excluded because of loss to follow-up. Seventeen second eyes were excluded, leading to a remaining number of 66 eyes of 66 patients that were used for analysis. Figure S1 illustrates the progress of patients throughout the five-year follow-up period. Patient characteristics and descriptives are summarized in Table 1. All patients were of Caucasian origin. The large majority had been diagnosed with POAG and a minority with pigmentary glaucoma. Most patients had advanced visual field loss and used three or more IOP-lowering medications.

**Table 1:** Baseline characteristics and demographics (*N* = 66).

Age, *years*, mean ± SD	65 ± 12
Gender, male (*n*)	52% (34)
Eye, right (*n*)	50% (33)
CCT, *µm*, mean ± SD	532 ± 42
CDVA, *LogMAR*, mean ± SD	0.18 ± 0.42
Pseudophakic (*n*)	35% (23)
Type of glaucoma (*n*)	
POAG	88% (58)
Pigmentary glaucoma	12% (8)
Glaucoma severity (*n*)	
Mild (MD, 0 to –6 dB)	27% (17)
Moderate (MD, –6 dB to –12 dB)	30% (19)
Advanced (MD, <–12 dB)	43% (27)
IOP, *mmHg*, mean ± SD	21.8 ± 4.2
No. of IOP-lowering medication, mean ± SD	2.5 ± 1.4
No. of IOP-lowering medication (*n*)	
0	14% (9)
1–2	32% (21)
≥3	55% (36)
Use of oral carbonic anhydrase inhibitors (*n*)	11% (7)
History of laser trabeculoplasty (*n*)	38% (25)

*CCT* central corneal thickness, *CDVA* corrected distance visual acuity, *IOP* intraocular pressure, *LogMAR* logarithm of the minimal angle of resolution, *MD* mean deviation, *POAG* primary open angle glaucoma, *SD* standard deviation.

### Effectiveness

**Fig. 1:**
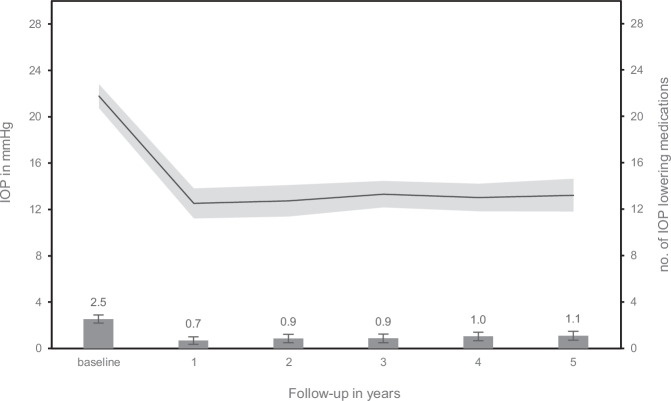
Estimated marginal mean intraocular pressure (IOP) and mean number of IOP-lowering medications before and after MicroShunt implantation. Error bars and shaded area represent the 95% confidence interval.

Table 2 presents observed means, and estimated marginal mean differences of IOP and the number of IOP-lowering medications during follow-up. IOP decreased significantly in comparison to baseline at 12 months. This reduction in mean IOP was sustained during follow-up (Fig. 1). The average number of IOP-lowering medications (95% confidence interval) was significantly reduced, from 2.5 (2.2–2.9) at baseline to 0.7 (0.3–1.0) at 1 year (*p* < 0.001). After the first year, medication use gradually increased up to 1.1 (0.7–1.5) at year 5 (*p* = 0.005).

**Table 2:** Intraocular pressure and number of IOP lowering medication after MicroShunt implantation during follow-up.

Visit	IOP (in mmHg)		IOP-lowering medication	
	observed means (SD)	estimated mean differences (95%-CI)	observed means (SD)	estimated mean differences (95%-CI)
Baseline	21.8 (4.2)	–	2.5 (1.4)	–
Year 1	12.5 (4.7)	–9.3 (–7.7 to –10.8)*	0.7 (1.2)	–1.9 (–1.4 to –2.3)*
Year 2	12.8 (5.1)	–9.0 (–7.5 to –10.5)*	0.9 (1.3)	–1.7 (–1.2 to –2.1)*
Year 3	13.3 (4.0)	–8.5 (–7.0 to –10.0)*	0.8 (1.3)	–1.7 (–1.2 to –2.1)*
Year 4	13.1 (4.1)	–8.7 (–7.2 to –10.3)*	1.0 (1.2)	–1.5 (–1.1 to –1.9)*
Year 5	13.1 (5.0)	–8.5 (–7.0 to –10.1)*	1.0 (1.2)	–1.4 (–1.0 to –1.9)*

Values are presented in observed means (standard deviation) and estimated mean differences (95% confidence interval). Estimated mean differences are based on estimated marginal means in comparison to baseline. Data was censored after additional glaucoma intervention. *p < 0.001 compared with baseline values.

*CI* confidence interval, *IOP* intraocular pressure, *SD* standard deviation.

**Fig. 2:**
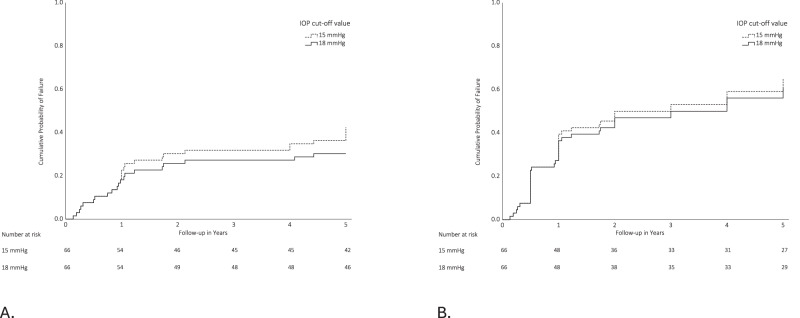
Kaplan–Meier survival curves. Kaplan–Meier survival curves showing the probability of failure according to qualified. **A** and complete (**B**) success definitions through 5 years after MicroShunt implantation. Failure was defined as an inadequate IOP control at two consecutive visits after 3 months of follow-up. Loss of light perception or additional glaucoma surgery were also classified as failures. Upper IOP values are 15 mmHg (dashed line) and 18 mmHg (solid line). If the IOP level was achieved without IOP-lowering medication, it was considered complete success (**A**). If IOP-lowering medication was used it was considered qualified success (**B**). The numbers of patients at risk at each follow-up visit are shown below each plot.

Time to failure data are presented for qualified and complete success for both cut-off values using Kaplan-Meier curves (see Fig. 2A, B, respectively). Qualified success, defined as an IOP level of 18 mmHg or less, despite the use of IOP-lowering medication, but without additional glaucoma interventions was achieved in 82% at 1 year, decreasing to 70% after 5 years. Using the most strict criterion, defined as complete success with an IOP level of 15 mmHg or less without the use of IOP-lowering medication, success was achieved in 61% after the first year, decreasing to 35% after 5 years.

### Postoperative interventions

Postoperative interventions after MicroShunt implantation are shown in Table 3. In seven eyes (11%) bleb needling was performed, 1.6 (1.2–3.9) months (median (min—max)) after surgery, whereas five eyes (8%) underwent bleb revision, 6.2 (1.8–19.8) months after surgery.

**Table 3:** Summary of IOP-lowering procedures and complications after MicroShunt implantation.

Glaucoma interventions	
Postoperative bleb management	
Bleb needling	10.6% (7)
Bleb revision	7.6% (5)
Additional glaucoma intervention	
MicroPulse cyclophotocoagulation	9.1% (6)
Trabecular bypass stent	3.0% (2)
Glaucoma filtration device	12.1% (8)
Trabeculectomy	4.5% (3)

Complications	
Intraoperative complications	
Dislocated IOL	1.5% (1)
Mild hyphaema	3.0% (2)
Early postoperative complications	
Epithelial defect	1.5% (1)
Mild hyphaema	12.1% (8)
Numerical hypotony ≤5 mmHg	28.8% (19)
Shallow AC requiring reformation	4.5% (3)
Wound leak (conservative treatment)	1.5% (1)
Choroidal detachment (conservative treatment)	4.5% (3)
Late postoperative complications	
Numerical hypotony ≤5 mmHg	–
Tube occlusion by iris*	3.0% (2)
Migration of stent	–
Tube erosion (conjunctiva)	1.5% (1)
Endophthalmitis	–

Data are presented in % (no.). Late postoperative complications are considered >3 months after the surgery. Data were censored after an additional IOP-lowering procedure. *Requiring reposition.

(*N* = 66).

*AC* anterior chamber, *IOL* intraocular lens.

Eleven out of the twelve eyes (92%) that underwent bleb needling or revision required further IOP-lowering interventions. If further IOP-lowering was required, it was performed 2.7 (0.2–10.8) months after the first bleb intervention. Four eyes (33%) underwent micropulse transscleral cyclophotocoagulation (micropulse—TSCPC) and seven eyes (58%) underwent further glaucoma filtration surgery to control IOP

In total, 19 eyes (29%) (including patients that previously underwent bleb intervention) required an additional IOP-lowering procedure: micropulse - TSCPC (9%), a glaucoma filtration device (12%), trabeculectomy (5%), or a trabecular bypass stent (3%).

Eighteen of the 42 phakic eyes (42%) underwent cataract surgery after implantation, with a median time between surgery and phacoemulsification of 26 months (range 9 to 51 months).

### Safety

Early hyphaema was commonly reported but was usually mild and self–limiting (Table 3). A single measurement of numerical hypotony at day one and/or week one was seen in 19 eyes (29%). Three eyes (5%) needed reformation of the AC with the aid of a viscoelastic agent in the first postoperative week. Three cases (5%) of (non-kissing) choroidal detachment in combination with hypotony were observed at week one, resolving spontaneously. After one month, hypotony had resolved in all cases. In two cases (3%), the iris occluded the lumen of the tube due to malposition of the MicroShunt and repositioning was successfully performed in the operating room in combination with an additional IOP-lowering procedure (cataract surgery combined with placement of trabecular bypass stent). In one case (2%), a conjunctival tube erosion occurred after surgical revision, after which the shunt was removed.

Using our definition of visual recovery, visual recovery occurred at one week in 73% increasing to 95% at three months. Persistent loss of more than 2 VA lines at the end of follow-up was seen in 2 eyes (3%), probably caused by progression of a pre-existent central scotoma. A decrease in VA to light perception or less did not occur. Mean deviation of the visual field did not increase significantly over time with an mean deviation of the visual field of –11.0 (–12.8 to –9.2) dB at baseline and –12.8 (–15.3 to –10.3) dB at 5 years (*p* = 0.364).

## Discussion

This study shows the long-term results, after five-year follow-up, with the MicroShunt in a single centre. Although this was a new procedure at the time, the results show that effective long-term IOP-lowering, with a mean IOP in the low teens, can be obtained after MicroShunt implantation with 0.2 mg/ml mitomycin C with a duration of two or three minutes, in patients with open-angle glaucoma. Furthermore, the average number of IOP-lowering medications was significantly reduced. Surgical results demonstrated a one-year qualified success rate, defined as an IOP level of 18 mmHg or less, regardless of the use of IOP-lowering medication, but without additional glaucoma interventions, of 82%, decreasing to 70% after five years. Nineteen eyes (29%) required an additional IOP-lowering intervention (micropulse-TSCPC, filtration surgery, or a trabecular bypass stent combined with cataract surgery). Any adverse events were usually mild and self-limiting and included early hypotony, shallow anterior chamber, and hyphaema. Long-term complication rates were low.

These results corroborate the sustained IOP reduction found in previous studies [22–24]. Mean IOP varied from 11.9 to 14.4 mmHg after two years of follow-up [10, 11, 24, 25]. After five years of follow-up, Batlle et al. reported a mean IOP of 12.4 mmHg [18]. However, it is challenging to compare success rates across studies due to varying follow-up periods, different MMC protocols used, and various definitions of surgical success. Bleb needling rates (11%) were lower than in previous studies (25%) [11]. Following our experience, a shift towards favouring open revision over bleb needling was noted, as the results of aqueous outflow from the tube appeared more favourable after revision. There is debate about choosing needling or open revision when implantation does not yield the expected results. According to a recent consensus paper open revision with the release or removal of scar tissue is preferred [26]. The present results show that most failures occurred within the first postoperative year. There might be a learning curve involved, as we often opted for direct further surgical interventions in earlier cases due to unfamiliarity with the device. This potential bias for reoperation may explain the higher intervention rates compared to other recent studies [18, 23]. A gradual increase in the use of IOP-lowering medications over time was also found, which aligns with results after trabeculectomy and other glaucoma filtration devices [27–29].

The complete success rates for IOP thresholds of 15 mmHg and 18 mmHg were nearly the same. This is in line with the achieved results of mean IOP of around 13 mmHg. As most patients had advanced glaucoma, we considered a postoperative IOP exceeding 15 mmHg as too high. Consequently, if the postoperative IOP raised above the threshold of 15 mmHg, IOP-lowering medications or interventions were commenced, which led to failure according to the complete success criteria. To mitigate potential bias and assess if in these cases complete success at an IOP target of 18 mmHg could have been achieved, wash-out of postoperative IOP-lowering would have to be performed at follow-up visits. However, due to the retrospective nature of this study, this was not feasible. The majority of patients in our cohort were using three or more classes of IOP-lowering medications prior to surgery. A sustained reduction in the number of IOP-lowering medications was achieved, which likely will have improved quality of life. However, this cannot be assessed under the current WGA-recommended definition of success.

Complications were mainly limited to early and self-limiting hypotony and some cases of choroidal detachment, all resolving with conservative treatment. Persistent hypotony did not occur. Our cohort showed one case of conjunctival tube erosion in a patient with an inflamed bleb, after an open revision was attempted. Conjunctival MicroShunt erosions have been described earlier [30, 31] and should be considered during follow-up, especially after an open revision. Due to this study’s retrospective nature, we did not include endothelial cell count; nonetheless, no cases of corneal decompensation were observed. Endothelial cell loss after MicroShunt implantation has previously been reported and investigated, showing higher rates of endothelial cell loss related to the tube-endothelium distance [32, 33]. Panarelli et al. showed similar endothelial cell loss two years after MicroShunt implantation and trabeculectomy in a comparative clinical trial [11].

No consensus has yet been reached on the optimal concentration of MMC. MMC is known to be effective in modulating the proliferative phase of wound healing in trabeculectomy, showing better IOP outcomes but at the cost of more complications, such as avascular blebs [34, 35]. However, even for trabeculectomy, no compelling evidence supports one MMC regimen over another. Even so, the most popular dosage for trabeculectomy augmentation remains 0.4 mg/ml during a 2-minute sponge application [36]. For the MicroShunt procedure, some evidence suggests that a 0.4 mg/ml dose will have a higher success rate than a 0.2 mg/ml dose [10, 12, 18, 23, 37]. This might be particularly relevant for non-Caucasian patients as these patients have a higher risk for fibrosis and scarring of the filtering bleb. A possible explanation for the improved results with a higher MMC concentration is the more posterior location of the filtering bleb compared to trabeculectomy, with a thicker Tenon’s capsule at this site. However, current methodologic restrictions require further evidence to establish the ideal dose of MMC use.

Further research is required to investigate which subgroups best suit this implant. To our knowledge, no data have been published on MicroShunt implantation in patients with pigmentary glaucoma. In our cohort, 12% of patients had pigmentary glaucoma. To date, some published cohorts included pigmentary glaucoma patients, but no studies have been published on the efficacy of the MicroShunt in PG in comparison to other types of glaucoma. A European multicentre study did not find significant differences between POAG and PEXG in IOP-lowering efficacy [13], which was supported by Nobl et al. [14]. Our cohort included open-angle glaucoma (primary open-angle glaucoma and pigmentary glaucoma). Pseudoexfoliative glaucoma did not occur in this cohort. Other forms of secondary glaucoma (e.g., eyes that previously underwent glaucoma surgery) did not occur. However, some evidence shows successful results after MicroShunt implantation for treating complex glaucoma cases such as refractory glaucoma and secondary glaucoma [15]. It also remains a subject of debate as to whether pseudophakia is a risk factor for failure after filtration surgery. A recent study with five years follow-up suggested that the order of trabeculectomy and cataract surgery did not differ significantly in success outcomes [38]. However, the study of Nguyen et al. demonstrated that patients who underwent phacoemulsification after trabeculectomy had a significantly increased risk of failure within one year after surgery compared to trabeculectomy in pseudophakic eyes [39]. In an expert consensus paper on the use of the MicroShunt, panellists agreed that to optimise surgical outcomes, patients should not have had phacoemulsification at least six months prior to MicroShunt implantation [40].

We found a 42% cataract surgery rate in phakic eyes, consistent with rates reported following other glaucoma surgery, such as filtration devices and trabeculectomy [4, 41, 42]. Glaucoma surgery was found to be associated with a higher incidence and progression of cataract [43]. In our cohort, this rate may be partially explained by pre-existing cataract and prioritising glaucoma surgery (MicroShunt implantation) over cataract surgery. Future research should explore the feasibility and outcomes of a combined MicroShunt and cataract procedure to possibly optimize management for these patients with pre-existing cataract.

A proposed advantage of MIGS and less invasive bleb-forming surgery is rapid visual rehabilitation [44]. After trabeculectomy, transient vision loss is common, and rehabilitation can sometimes take up to two years [45]. As there is no benchmark for visual rehabilitation, we proposed a definition of a LogMAR CDVA of one and a half lines within baseline CDVA, as limits of agreement for test-retest measures of visual acuity were shown to be about ± 0.15 logMAR [20]. When applied this definition, the majority of patients achieved visual recovery after the first week, and most patients recovered within three months after surgery, similar to the results of Panarelli et al. [11], endorsing the proposed advantage for earlier visual recovery after less invasive surgeries.

Certain limitations need to be acknowledged. Firstly, this was a single-centre study with a relatively small sample size, which limits its external validity. Secondly, this is a noncomparative case series and does not provide information on how this device works compared to traditional glaucoma surgery such as trabeculectomy. Notwithstanding these points, this study has several notable strengths. Real-world data over such a long period contribute to important insights into the use of the device in daily clinical practice. Additionally, the reported results confirm the sustained safety and effectiveness of this new implant over the long term. Further randomised studies comparing traditional glaucoma surgery with this procedure are mandatory.

As most new glaucoma devices are costly, there is also a need for cost-effectiveness studies that compare MicroShunt implantation with the ‘gold standard’ trabeculectomy. Currently, randomised controlled (cost-) effectiveness studies are ongoing worldwide [46].

In conclusion, the present study demonstrates a long-term sustained reduction in mean IOP and number of IOP-lowering medications with the MicroShunt in a Caucasian population with fairly advanced glaucoma. The procedure was found to be safe, with no significant long-term complications and a very low rate of implant erosion. However, nearly one-third of patients in this cohort required further IOP-lowering surgery. Further research is warranted to identify which subgroups of glaucoma patients would benefit most from this implant and to determine the optimal concentration of MMC for achieving the best outcomes.

Supplemental material is available at Eye’s website.

## Summary

### What was known before

- The PreserFlo MicroShunt has been introduced to offer a less invasive bleb forming alternative to traditional filtering surgery for glaucoma.
- Long-term evidence on the PreserFlo MicroShunt is limited.

### What this study adds

- In this study we report on our 5-year outcomes of PreserFlo MicroShunt implantations in patients with open-angle glaucoma.
- The PreserFlo MicroShunt was found to be a safe procedure, leading to a sustained reduction in mean IOP and number of IOP-lowering medications.

## Supplementary information

Supplementary Figure 1

Supplementary Figure 1 Legend

## Data Availability

Due to privacy and confidentiality agreements, the dataset is not publicly available. However, de-identified data may be made available upon reasonable request, subject to approval by the relevant ethics committee and institutional review board.
