# Supplementary material for: Five-year follow-up with the PreserFlo MicroShunt for open-angle glaucoma
Source: Eye (Lond). 2025 Feb 20;39(8):1540–6. doi: 10.1038/s41433-025-03707-3 (PMC12089333; doi:10.1038/s41433-025-03707-3)

Supplementary Figure 1. Flow chart of patient progress and reasons of study discontinuation during follow-up.

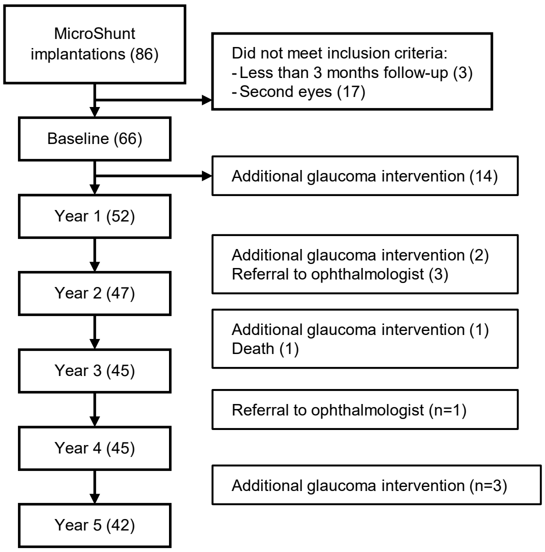

Supplement: Supplementary file 1 — Supplementary Figure 1 [file 41433_2025_3707_MOESM1_ESM.pdf]
